# Noise Suppression in Compressive Single-Pixel Imaging

**DOI:** 10.3390/s20185341

**Published:** 2020-09-18

**Authors:** Xianye Li, Nan Qi, Shan Jiang, Yurong Wang, Xun Li, Baoqing Sun

**Affiliations:** 1 Institute of Marine Science and Technology, Shandong University, Qingdao 266237, China; xianyeli@mail.sdu.edu.cn; 2 School of Information Science and Engineering, Shandong University, Qingdao 266237, China; shanjiang0903@163.com (S.J.); yrw@sdu.edu.cn (Y.W.); baoqing.sun@sdu.edu.cn (B.S.); 3 Department of Electrical and Computer Engineering, McMaster University, Hamilton, ON L8S 4k1, Canada; lixun@mcmaster.ca

**Keywords:** single-pixel imaging, detection noise suppression, compressive sensing

## Abstract

Compressive single-pixel imaging (CSPI) is a novel imaging scheme that retrieves images with nonpixelated detection. It has been studied intensively for its minimum requirement of detector resolution and capacity to reconstruct image with underdetermined acquisition. In practice, CSPI is inevitably involved with noise. It is thus essential to understand how noise affects its imaging process, and more importantly, to develop effective strategies for noise compression. In this work, two ypes of noise classified as multiplicative and additive noises are discussed. A normalized compressive reconstruction scheme is firstly proposed to counteract multiplicative noise. For additive noise, two types of compressive algorithms are studied. We find that pseudo-inverse operation could render worse reconstructions with more samplings in compressive sensing. This problem is then solved by introducing zero-mean inverse measurement matrix. Both experiment and simulation results show that our proposed algorithms significantly surpass traditional methods. Our study is believed to be helpful in not only CSPI but also other denoising works when compressive sensing is applied.

## 1. Introduction

In traditional photography, denoising is a postprocess of image acquisition that employs algorithms to suppress noises involved with signals in recorded images. In other words, little can be done to reduce existing noise throughout the stage of image acquisition. Recent advantages on computational imaging have changed traditional photography by introducing modulation approaches during image recording, which enable extra operations to enhance the acquisition efficiency, including noise suppression. Among various computational imaging schemes, compressive imaging is well known for its capacity to restore images through undersampling data using compressive sensing (CS) theory [1,2]. Compressive imaging takes advantage of the sparsity of natural images to retrieve spatial information below the Nyquist sampling limit [3]. It is widely discussed for its applications to break through the limitation of imaging facilities [4] or even the detection mechanism itself [5]. These applications cover a variety of areas including radar detection [6], single-pixel three-dimension imaging [7], ultrafast imaging [8], snapshot spectral imaging [9], spectrum retrieval [10], optical information security [11], etc.

Among these applications, compressive single pixel imaging (CSPI) is a novel imaging scheme that combines advantages of both single pixel camera and CS. It restores images using nonpixelated detection and, meanwhile, reduces the number of measurement iterations for image reconstruction. Its minimum requirement for detector resolution offers great advantages at special wavelength when focal plane detector arrays are expensive or technically unreachable. In a practical CSPI experiment, a spatial light modulator (SLM) is used to modulate the image of an object for compressive sampling. All the light after modulation is then collected by a single-pixel detector (SPD). Both modulation and detection processes introduce noise to the signals and reduce the final imaging quality. Although the compressive algorithm can suppress random noise in the reconstruction process [12], this denoising process has a very limited effect. Fortunately, as a computational imaging scheme [13], modulation-based data acquisition and image reconstruction are separated, which means that it is possible to reduce noise experimentally before image reconstruction. However, most denoising works are based on traditional correlation single-pixel imaging [14,15]; for CSPI experiment, there is still a lack of systematic noise analysis and optimizations.

In this paper, we investigate the impact of different noise types on image quality and build a novel, yet simple evaluation model for excited physical noise. Meanwhile, though data acquisition strategies are improved to realize noise suppression in CSPI, this process is independent of traditional image denoising process and can also be applied to enhance imaging quality. In CSPI system, noise is mainly analyzed in two categories [16], multiplicative noise and additive noise. Multiplicative noise refers to stochastic distortions in modulation and detection procedures as the fluctuation of structured illumination. This issue has been discussed in normalized ghost imaging (NGI) [17], however, noise property in CSPI could be more complicated due to its strong correlations between each iteration. A normalized CSPI scheme is thus proposed to eliminate noise, which employs a reference detector and corresponding normalization approach. Additive noise, in general, is an additional term that is independent of measurement signals, which adds an unexpected bias term to original detection processes. Two classical iterative optimization processes—i.e., gradient descent and Newton’s method—are adopted to analyze the effect of additive noise. These methods have been widely applied in many compressive reconstruction solvers such as GraDes [18], TVAL3 [19], $L_{1}$-magic [20], and BFGS [21]. In particular, a detailed comparison between two typical solvers, $L_{1}$-magic and TVAL3, is implemented in both numerical simulations and experiments, and their performance under single and differential detections is discussed.

## 2. Compressive Imaging Theory

CSPI utilizes compressive sensing to reconstruct images from sub-Nyquist detections, which can effectively extended imaging dimensions through appropriate sparse transform, for instance, in time, space, and spectrum. Generally, CSPI scheme can be regarded as a linear detection procedure, and mathematically described as

(1) y=Φx,

where $\Phi \in R^{M\times N}\left(M\ll N\right)$ is the measurement basis or sensing matrix, $x\in R^{N}$ is the column vector representing the object transmission function, and $y\in R^{M}$ is the ideal compressed signal containing *M* entries. The sampling ratio is defined as M/N. If the signal x is sparse or can be sparse in a specified transform domain and sensing matrix satisfies the restricted isometry property (RIP) [22], the object can be recovered by solving an optimization problem described as

(2) $$\tilde{x}=\underset{x}{\arg\min}\frac{1}{2}{∥y-\Phi x∥}_{2}^{2}+\lambda \Psi \left(x\right),$$

where Ψ is a regularization term that constrains the sparseness of x, and λ is a Lagrange multiplier. In compressive imaging, total variation [23] and dictionary learning [24] are widely used to realized the sparseness constraint in reconstruction procedure.

In signal processing theory [16], noise in CSPI can be mainly categorized as multiplicative noise and additive noise. Generally taking these two types of noise into account, the sampling process with noise is then expressed as

(3) $$\hat{y}=l*\Phi x+n,$$

where $l\in R^{M}$ represents the multiplicative noise with *M* fluctuation index and the operator ‘∗’ represents elementwise product operation. n as a bias term denotes additive noise in *M* detections. In CSPI experiment, multiplicative noise is mainly caused by fluctuation or flicker of illumination source or modulation devices; and additive noise involves factors such as background light and detector noise.

## 3. Multiplicative Noise

**Figure 1:**
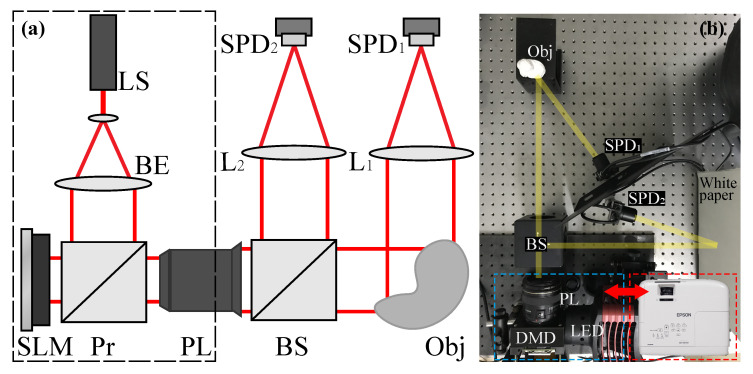
(**a**). The schematic diagram for normalized ghost imaging (NGI). The light source (LS) is expanded through a beam expander (BE). A structured light is generated through a spatial light modulator (SLM) and projected on the object trough a project lens (PL). Then, the structured light is divided into two arms by a beam splitter (BS). The object beam is detected by a single-pixel detector (SPD${}_{1}$) after the modulation of the object (Obj). The intensity in reference arm is directly detected by another SPD (SPD${}_{2}$). L${}_{1}$–L${}_{2}$: lens; Pr: prism. (**b**). Practical experiment arrangements for multiplicative noise (EPSON projector) and additive noise (DMD projector) experiments. The rough white paper in reference arm is employed to homogenize the reference light.

To remove the effect of fluctuation of structured illumination, a reference beam is added to apply normalization measurement. The schematic diagram of NGI is shown in Figure 1. A light source is modulated by a SLM and split into two arms by a beam splitter. The object beam illuminates the object and is then collected by a SPD (SPD${}_{1}$). The reference beam is detected by another SPD (SPD${}_{2}$) directly. Normalization detection values $\hat{y}$ in NGI can be calculated through

(4) $${\hat{y}}_{i}=\frac{s_{i}}{r_{i}},$$

where $s_{i}$ and $r_{i}$ are detection values for the object beam and reference beam in *i*th measurement iteration, respectively. Considering the influence of multiplicative noise, $s_{i}$ can be expressed as $l_{i}y_{i}$, where $l_{i}$ is the illumination fluctuation determined by both the light source and the modulator, and $y_{i}$ is the ideal detection value in object beam under stable illumination. The numerator $r_{i}$ is equal to $l_{i}p_{i}$, where $p_{i}$ is the sum of entries in *i*th row of the measurement matrix Φ, which can be considered as total transmission of the coded pattern or hologram energy in *i*th measurement. Therefore, the multiplicative fluctuation can be easily canceled out through normalized operation.

However, the construction of coded patterns in compressive imaging are not always energy keeping, indicating that $p_{i}$ can also introduce noise in normalization detections. To illustrate the influence of $p_{i}$, we further rewrite Equation (4) so that

(5) $$\begin{array}{cc} {\hat{y}}_{i} & =\frac{y_{i}}{p_{i}}\\ \; & =y_{i}+\delta_{i}y_{i}, \end{array}$$

where $\delta_{i}=\left(1-p_{i}\right)/p_{i}$. $\delta_{i}y_{i}$ can be regarded as an error introduced due to the fluctuation of $p_{i}$. This error term is negligible in iterative NGI, as it is mostly canceled out after the whole averaging process. In CSPI however, it cannot be ignored and could lead to complete reconstruction failure. This issue is hard to eliminate when the random diffuser [25] or shifted mask [26] is applied to generate the structured patterns. Therefore, to guarantee solid reconstruction, power variation caused by the fluctuation of $p_{i}$ in reference arm needs to be postprocessed. Hence, we propose a normalized scheme for CSPI (NCSPI) which is expressed as

(6) $$y_{i}=\frac{s_{i}p_{i}}{r_{i}}.$$

**Figure 2:**
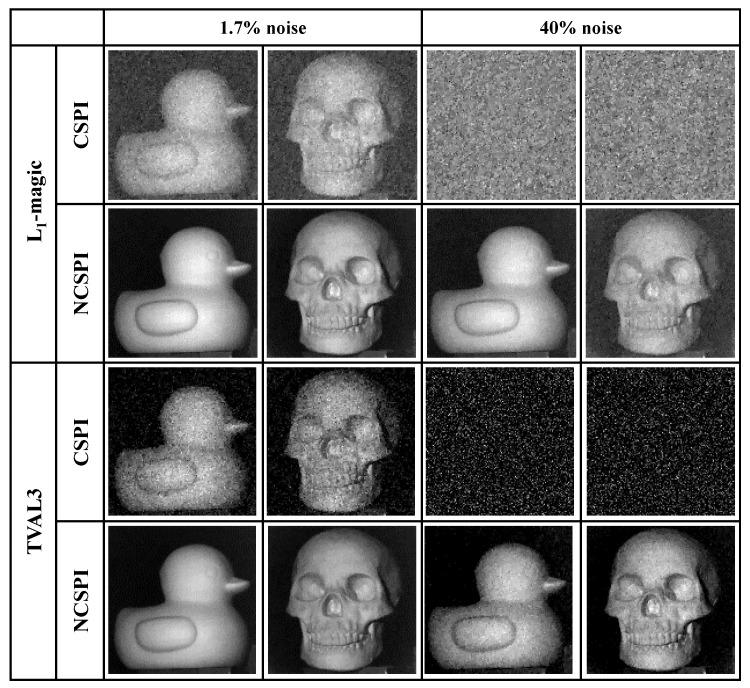
Experimental comparison between compressive single-pixel imaging (CSPI) and normalized CSPI (NCSPI) with 1.7% fluctuation and 40% artificial noise, respectively. Two classical reconstruction solvers, $L_{1}$-magic and TVAL3, are adopted to retrieve final results. The spatial resolution is set at 128×128 pixels and 8000 times differential detections (48.8% sampling ratio) are realized to reconstruct every image.

The experiment is then conducted to examine the feasibility of NCSPI. A light projector (EPSON CH-TW750) with an ultra-high-pressure (UHP) mercury lamp and liquid-crystal SLM is employed as the structured illumination source. Two SPDs (Thorlabs PDA-100A) are employed to detect the object and reference beams simultaneously. To minimize unwanted additive noise, differential detection is applied [27]. To measure the level of projection fluctuations, a piece of white paper is placed in the object plane. Then, an all-white pattern is projected and ten thousand intensity values are acquired from SPD${}_{1}$ continuously. In each measurement, the detection intensity is acquired form the mean value of 10,000 detection values, corresponding to a sampling frequency of our data acquisition device of 200 kHz. Then, the noise ratio can be calculated as

(7) $$\eta =\frac{\langle \left|d-\langle d\rangle \right|\rangle }{\langle d\rangle }\times 100\%,$$

where d is the detected intensity sequence and the operation ‘$\langle \cdot \rangle$’ represents the averaging operation. The fluctuation of the projector illumination output is measured as 1.7% at its normal working mode. It is under this mode that our first set of reconstructions is carried out. Two typical compression reconstruction solvers $L_{1}$-magic (TV minimization, $L_{1}$ norm, and Newton’s method) and TVAL3 (TV minimization, $L_{1}$ norm, and steepest descent method) are used and their results are shown in Figure 2. Reconstruction is conducted at a resolution of 128×128 pixels with 8000 measurement iterations. In each measurement iteration, a random binary pattern is projected and the projection area is set as about 25 cm × 25 cm. Therefore, the size of our measurement matrix is 8000×16384, which is generated from a uniformly distributed pseudorandom integers generator “randi()” in MATLAB. We directly check its rank to ensure the matrix we generated is row full rank. All the reconstruction procedures are implemented using MATLAB R2018a (Intel-i9900K, 32GB RAM, NVIDIA RTX-2080Ti) and corresponding results are shown in Figure 2. Distinctly, NCSPI presents a much better improvement in image quality. To further elaborate this improvement, a second set of experiments is carried out by adding artificial random fluctuations. Before the pattern is projected, we randomly generate a illumination factor $l_{i}$ to control the grayscale of projected pattern, this factor can be seen as the multiplicative noise factor, as it in Equation (3), and influences the brightness of projected light. The artificial noise is measured as about 40% of the averaging illumination intensity. While in the reconstructions, traditional CSPI reconstruction fails to generate images, the normalized schemes generate good images under both solvers. Our results show that NCSPI can effectively eliminate the influence of multiplicative noise in CSPI.

**Figure 3:**
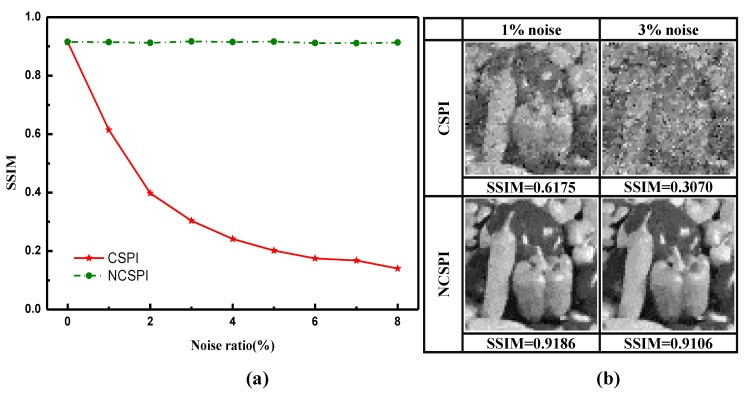
(**a**) The qualitative comparison for NCSPI and CSPI under different noise ratios using $L_{1}$-magic solver. The reconstruction resolution is set at 64×64 pixels. In simulation, 2000 times measurements are carried out, which means the compression ratio is about 48.8%. (**b**) The detailed reconstruction images and their SSIM indices under 1% and 3% noise ratios. NCSPI performs better in offsetting multiplicative noise and produces clearer images in both noise ratios.

To quantitatively analyze the performance of NCSPI, a simulation analysis is carried out, where illumination fluctuation is considered to be the single noise source. Structural Similarity Index (SSIM) is adopted to evaluate reconstruction quality. Figure 3a shows SSIM of NCSPI and CSPI under different noise levels. While SSIM of CSPI decreases with the increase of fluctuation, SSIM of NCSPI stays in a very stable level.

Summarily, in this section, conventional normalized scheme is improved by taking the energy variation of the pattern itself into the normalized procedure. This improvement ensures that our normalized scheme is suitable for CSPI. Both experiment and simulation results demonstrate that the proposed normalized method is very effective against multiplicative noise in CSPI. It is believed that our proposed method will provide a feasible scheme for noise suppression in passive light and low-light application scenarios; in these scenarios, the multiplicative noise is hard to be avoided but influences imaging quality distinctly.

## 4. Additive Noise

### 4.1. The Influence of Additive Noise

Additive noise in CSPI experiment mainly includes detection noise and background light noise. As discussed in Section 2, the process of SPI with additive noise can be expressed as

(8) $$\hat{y}=\Phi x+n.$$

**Figure 4:**
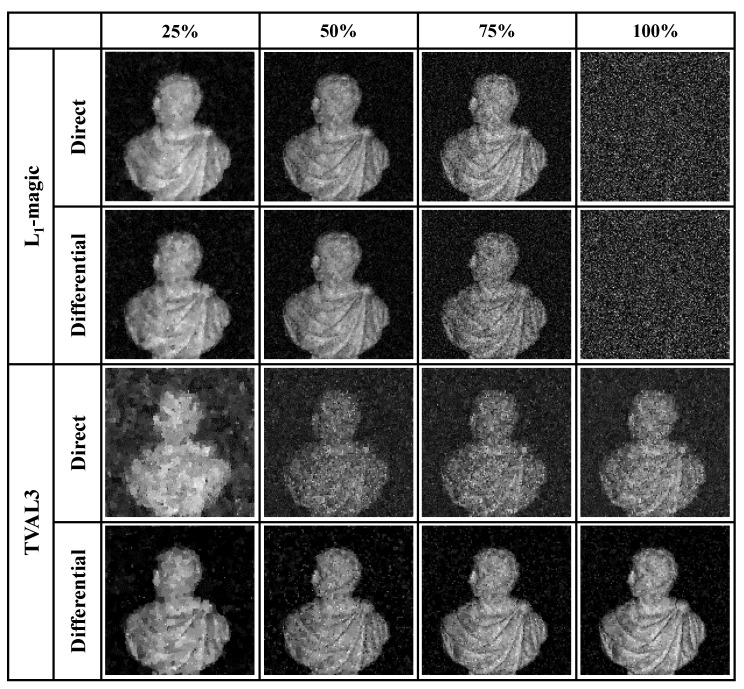
Experiment results for $L_{1}$-magic and TVAL3 algorithms using either direct or differential detection. Different sampling ratios (25%, 50%, 75%, and 100%) are displayed in column. The experiment resolution is set as 128×128 pixels.

Different from multiplicative noise, additive noise is hard to be eliminated by experimental schemes or algorithms. In general, the differential detection method is an intensively used way to suppress additive noises. A comparison experiment is thus carried out to show how differential detection improves CSPI. In the experiment, structured illumination is generated using a digital micromirror device (DMD, ViALUX V7000) and a white-light LED (Thorlabs MNWHL4) with stable illuminance, the detailed experiment arrangement is exhibited in Figure 1b. To minimize the effect of illuminance fluctuation, the normalization algorithm proposed in Section 3 is also adopted. The noise ratio of our experimental arrangement is also measured and calculated using the method proposed in Section 3, which is measured in a level equivalent to 1% of the average detection value. However, the detection numbers here are set as 50 detections and the sampling frequency is modified as 500 kHz. Fast modulation frame set as 20 kHz is implemented to project random binary patterns in 128×128 pixels, and the projection area is set as about 8 cm×8 cm. Both $L_{1}$-magic (TV minimization, $L_{1}$ norm, and Newton’s method) and TVAL3 (TV minimization, $L_{1}$ norm, and steepest descent method) solvers are employed and compared in Figure 4. It is observed that unlike using TVAL3, where differential measurement reveals a better effect on imaging reconstructions, $L_{1}$-magic reconstruction presents no significant improvement. Moreover, in the extreme, $L_{1}$-magic with 100% sampling ratio even fails to generate an image.

### 4.2. Theoretical Explanations

To understand it, we look into details of the solvers. In $L_{1}$-magic, Newton’s method is employed at each log-barrier iteration to minimize the objective function. If we ignore the sparse regularization term in Equation (2) and consider the calculation in one iteration, Newton’s method can be simplified as

(9) $$\begin{array}{cc} x^{k+1} & =x^{k}-H_{x^{k}}^{-1}\nabla f\left(x^{k}\right)\\ \; & \approx {\left(\Phi^{T}\Phi \right)}^{-1}\Phi^{T}\hat{y}≐\Phi^{†}\hat{y}\\ \; & =\tilde{x}+\Phi^{†}n, \end{array}$$

where $\nabla f(x^{k})$ is the gradient of $f(x^{k})$ and $H_{x^{k}}$ is the Hessian matrix in *k*th iteration. The superscript *T* denotes the matrix transpose operation and $\Phi^{†}$ is pseudo-inverse of Φ [28]. $\tilde{x}$ is the reconstruction with no physical noise, and $\Phi^{†}n$ denotes the error term caused by physical noise. The mean square error (MSE) of the results can be calculated as

(10) $$\begin{array}{c} \mathrm{MSE}=\frac{1}{N}{\left(\Phi^{†}n\right)}^{T}\Phi^{†}n. \end{array}$$

Similarly, steepest descent method—which is employed in every iteration of TVAL3—can be simplified as

(11) $$\begin{array}{cc} x^{k+1} & =x^{k}-\nabla f\left(x^{k}\right)\\ \; & \approx \tilde{x}+\Phi^{T}n. \end{array}$$

Its corresponding MSE can be calculated as

(12) $$\begin{array}{c} \mathrm{MSE}=\frac{1}{N}{\left(\Phi^{T}n\right)}^{T}\Phi^{T}n. \end{array}$$

**Figure 5:**
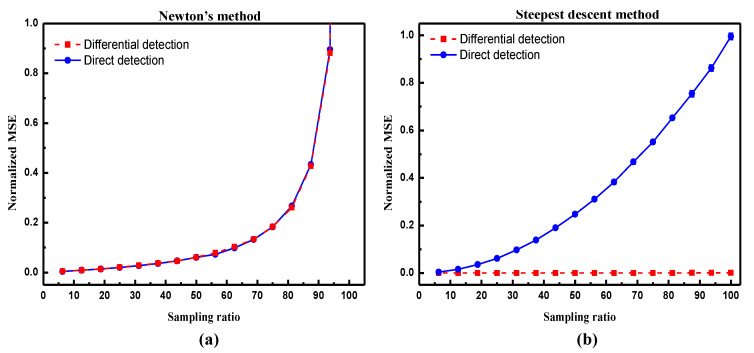
Normalized mean square error (MSE) curves calculated for one-step iteration in (**a**) Newton’s method and (**b**) steepest descent method with different sampling ratio, respectively. It is noted that as MSE in 100% sampling ratio in (**a**) is extremely large, MSE in 95% sampling ratio under direct detection is thus adopted to normalize the data.

To compare these two different methods, numerical calculation of MSEs are carried out under different sampling ratios, where Φ is a random binary matrix, as used in previous sections. Noise n is simulated as a non-negative term following a Gaussian-like distribution, which depends not only on the additive noise but also on the construction of $\Phi^{†}$ or $\Phi^{T}$, as indicated in Equations (9) and (11). Shown in Figure 5a, MSEs under direct detection and differential detection using $L_{1}$-magic both increase with sampling ratios. This is because in Newton’s iteration, the pseudo-inverse operation of sensing matrix Φ cannot guarantee zero-mean property, where the ‘zero-mean property’ indicates that the mean value of each row in the matrix is zero. The non-zero-mean property of the pseudo-inverse matrix causes the noise accumulation with the increase of sampling numbers—that is the reason why the worst results may appear under large sampling ratios. However, as exhibited in Figure 5b, differential measurement using TVAL3 solver gives a clear suppression of MSE, as the transpose operation in steepest descent method keeps the zero-mean property of sensing matrix and thus reduces the noise.

**Figure 6:**
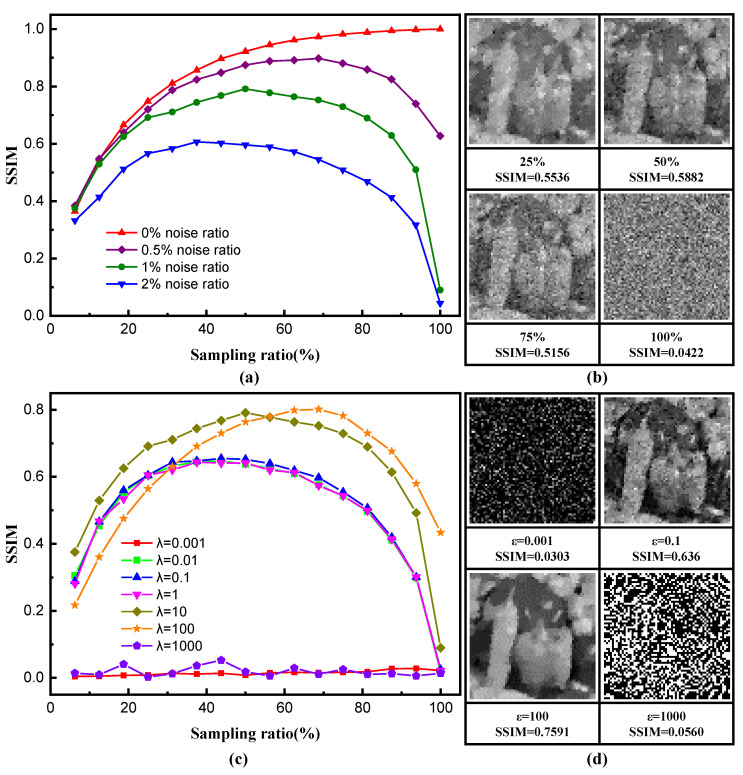
(**a**) The structural similarity index (SSIM) curves for reconstructed images at different sampling ratio under different noise ratios, the parameter ϵ for these results is set as 10. (**b**) The detailed images reconstructed from different sampling ratios under 2% noise ratio. (**c**) The SSIM curves reconstructed using different ϵ values under 1% additive noise ratio. (**d**) The detailed reconstructed images at 50% sampling ratio. The reconstruction resolution is set at 64×64 pixels.

Depicted in Figure 6a, the dashed curve at the top indicates reconstruction using $L_{1}$-magic algorithm with no physical additive noise, which corresponds to the reconstructed $\tilde{x}$. As sampling ratio increases, better reconstruction is observed with larger sampling ratio. However, as seen in Figure 5a, MSE from Newton’s method increases fast, even approaching an extremely high value, indicating that at certain point, the influence of noise overruns the contribution of reconstruction. This is further supported by reconstructions with three different additive noise ratios in terms of SSIM, plotted in Figure 6a. With the increase of additive noise, the reconstruction quality becomes worse and worse; meanwhile, the error reconstruction phenomenon at high sampling ratio is more and more evident. It can also be observed that as MSE becomes extremely large at 100% sampling ratio, imaging reconstruction eventually collapses, as shown in Figure 6b. Besides, the multiplier λ in Equation (2) is an important parameter to relax the detection constraint under noisy environments. In $L_{1}$-magic solvers, a predefined parameter ϵ can be seen as a equivalent parameter of λ. Figure 6c shows the SSIM curves reconstructed using different ϵ under 1% additive noise ratio. Apparently, a large ϵ can indeed reduce or even eliminate (it depends on the noise ratio) the error reconstruction phenomenon at high sampling ratio, but still cannot eliminate the influence of additive noise. More importantly, as shown in Figure 6d, due to the increased weight of the regularization term, the large ϵ can lead to the loss of image edge detail information, and even to reconstruction failure.

In fact, pseudo-inverse solutions in SPI are well known to be extremely sensitive to noise [29], and some works also have provided some mitigation techniques such as orthogonalization or new calculation methods [30,31,32]. However, it is still the first study as far as we know to analyze the noise trend with increased sampling ratio for CSPI, when different detection methods and optimization methods are applied. Moreover, this phenomenon could happen in other algorithms or solvers that also adopt Netwon’s method or least square calculation in iteration processes, such as OMP and SAMP algorithms [33,34], and even becomes exaggerated in absence of iterative optimization processes [35].

### 4.3. Experimental Verifications and Discussion

**Figure 7:**
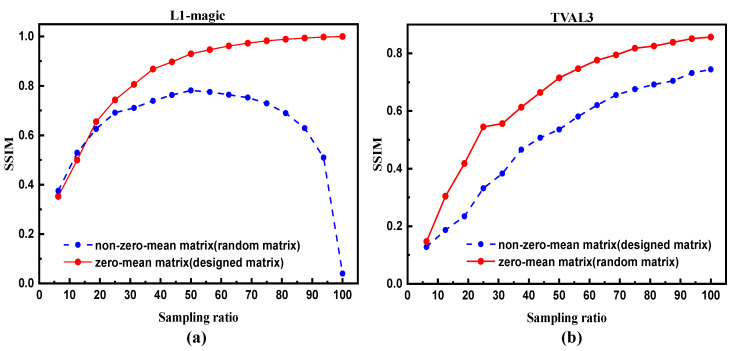
Reconstruction results from two designed matrices. (**a**) The comparison of reconstruction results from the designed zero-mean matrix $\Phi_{1}$ and a random matrix; 1% additive noise is simulated and $L_{1}$-magic solver is employed. (**b**) The comparison of reconstruction results from another designed matrix $\Phi_{2}$ and a random matrix; 1% additive noise is simulated and TVAL3 solver is employed. The reconstruction resolution is set at 64×64 pixels.

To examine our argument of the significance of zero-mean property preservation, a matrix $\Phi_{1}$ is purposely designed whose pseudo-inverse follows a zero-mean distribution, we get this matrix through calculating the pseudo-inverse of a zero-mean binary matrix ($\left\{-1,1\right\}$, uniform probability distribution). It is known that since additive noise satisfies a Poisson-like distribution, this designed matrix can effectively offset additive noise when $L_{1}$-magic is adopted. Shown in Figure 7a, the solid curve, representing SSIM under 1% noise ratio reconstructed by this designed matrix, exhibits a big improvement compared to the one using a random binary matrix, even when the sampling ratio is large. These results effectively prove the feasibility of our noise model, and also indicate a latent noise suppression method.

In contrast, another special matrix $\Phi_{2}$, whose transposition distribution does not follow zero-mean property is constructed. In our example, the mean value of each column in $\Phi_{2}$ is between −0.6 and 0.6, we realize this by adjusting the proportion of elements 0 and 1 in each column of $\Phi_{2}$. We repeat the simulation under 1% additive noise using TVAL3 solvers. The SSIM of reconstruction results are plotted in Figure 7b. It is clear to see that non-zero-mean matrix behaves worse than the traditional random matrix. Therefore, the distribution of measurement matrix is a key factor in the reconstruction involved with additive noise. With differential measurement, steepest descent method can easily offset additive noise using a traditional random matrix due to its preservation of zero-mean property. For Newton’s method, however, zero-mean-inverse is prerequisite to achieve reasonable reconstructions.

Therefore, the SNR does not always decrease with the sampling ratio increase when Newton’s method is applied. In addition, differential detection is not always effective for steepest descent method. The key to suppress the additive noise is the zero-mean property of measurement matrix. It is believed that the proposed noise evaluation model can provide a new matrix design reference to reduce existing additive noise.

## 5. Conclusions

To conclude, the performance of compressive single-pixel imaging with both multiplicative noise and additive noise is discussed. Two typical compressive solvers, $L_{1}$-magic and TVAL3, are employed in experiment and simulation studies. For multiplicative noise, a normalized compressive reconstruction scheme is proposed which can effectively remove the imaging defection caused by factors such as the fluctuation of illumination. Both experiment and simulation results show that our normalization scheme works well in reducing multiplicative noise. Unlike multiplicative noise, additive noise such as detector noise is independent of sampled signals. Differential acquisition scheme can suppress additive noise, yet the efficiency of noise suppression varies depending on the adopted compressive algorithm. In the work, an interesting phenomenon using $L_{1}$-magic solver is found, showing that more measurement could lead to poorer reconstruction quality. It is because the reconstruction noise rises faster, especially when sampling ratio approaches to its full measurement, which, in essence, is attributed to the zero-mean property of sensing matrix.

Different from noise suppression in traditional digital image processing, our work reduces noise during data acquisition by experimental setup and patterns design, which is independent and complimentary to traditional image denoising algorithms. It is believed that our study provides an important guideline for the application of compressive sensing in not only single-pixel imaging but also other compressive imaging techniques when practical noise is involved. Future study will be focused on the development of CS denoising algorithm, particularly when nonzero mean matrices are involved; for example, in coherent diffraction imaging [36] and coded aperture compressive imaging [37], which have been widely applied in spectral analysis and biomedicine imaging. Other noise characteristics, such as noise distribution, also need to be considered.

## Figures and Tables

**Table:** 

**Table:** 

**Table:** 

**Table:** 

**Table:** 

**Table:** 

**Table:** 
